# Enhancing knowledge and coordination in obesity treatment: a case study of an innovative educational program

**DOI:** 10.1186/s12913-019-4119-9

**Published:** 2019-05-02

**Authors:** Tonje C. Osmundsen, Unni Dahl, Bård Kulseng

**Affiliations:** 1 NTNU Social Research, Dragvoll Allé 38b, N-7491 Trondheim, Norway; 2 Norwegian Hospital Construction Agency, Klæbuveien 118, 7031 Trondheim, Norway; 3 Centre for Obesity Research (ObeCe), Clinic of Surgery, St. Olavs University Hospital, 7006 Trondheim, Norway; 4 Department of Cancer Research and Molecular Medicine, Norwegian University of Science and Technology, N-7489 Trondheim, Norway

**Keywords:** Integrated care, Education, General practitioners, Specialist training, Obesity, Diabetes, Case study

## Abstract

**Background:**

Currently, there is a lack of knowledge, organisation and structure in modern health care systems to counter the global trend of obesity, which has become a major risk factor for noncommunicable diseases. Obesity increases the risk of diabetes, cardiovascular diseases, musculoskeletal disorders and cancer. There is a need to strengthen integrated care between primary and secondary health care and to enhance care delivery suited for patients with complex, long-term problems such as obesity. This study aimed to explore how an educational program for General Practitioners (GPs) can contribute to increased knowledge and improved coordination between primary and secondary care in obesity treatment, and reports on these impacts as perceived by the informants.

**Methods:**

In 2010, an educational program for the specialist training of GPs was launched at three hospitals in Central Norway opting for improved care delivery for patients with obesity. In contrast to the usual programs, this educational program was tailored to the needs of GPs by offering practice and training with a large number of patients with obesity and type 2 diabetes for an extended period of time. In order to investigate the outcomes of the program, a qualitative design was applied involving interviews with 13 GPs, head physicians and staff at the hospitals and in one municipality.

**Results:**

Through the program, participants strengthened care delivery by building knowledge and competence. They developed relations between primary and secondary care providers and established shared understanding and practices. The program also demonstrated improvement opportunities, especially concerning the involvement of municipalities.

**Conclusions:**

The educational program promoted integrated care between primary and secondary care by establishing formal and informal relations, by improving service delivery through increased competence and by fostering shared understanding and practices between care levels. The educational program illustrates the combination of advanced high-quality training with the development of integrated care.

**Electronic supplementary material:**

The online version of this article (10.1186/s12913-019-4119-9) contains supplementary material, which is available to authorized users.

## Background

Obesity and diabetes are grave international health problems [1, 2] and the economic burden for both patients and national economies is significant [3]. Obesity is a complex condition often involving several other chronic and serious diseases requiring lifelong follow-up [4–6]. It often affects whole families and intervention is needed at multiple levels, including social and psychological dimensions. Treatment is therefore complex and time-consuming and poses a challenge for the organisation of health care services [7, 8]. Currently, there is a lack of well-established integrated approaches for prevention and treatment across health care levels.

### Treatment offerings

To tackle these challenges, the coordination of services needs to be improved [7, 9, 10]. In Norway, the Coordination Reform was launched in 2010 [11]. Here, as in other countries [12], integrated care is accompanied by goals for moving patients from secondary health care to primary care, increasing focus on prevention and health-promoting activities and patient involvement. Obese and overweight patients with related diseases have traditionally been offered treatment in primary care, but in recent years the number of patients with severe obesity and complications has increased and consequently, so has the number of patients referred to secondary health care. To counter such a development, primary and secondary health care providers should jointly develop integrated care based on an understanding of the disease’s complexity. In addition, knowledge and capacity at the primary health care level must be improved and services targeted at prevention and cure must be made available.

Although health care workers are key players in the effort to stop the obesity trend in the population and to prevent the complications of obesity, international research suggests that obese patients do not receive adequate help for their health problems. Studies have shown that health care providers’ treatment and attitude towards overweight and obese patients are governed by poor knowledge and inconsistencies [13, 14] and have a strong weight bias, indicating stigma [15, 16]. A mapping in Central Norway showed a lack of knowledge and tools for how to treat and prevent overweight and obesity [17]. Less than half of the obese patients who sought medical help for their lifestyle problems were advised to lose weight by their GP [18] or given exercise counselling [19], and while research has shown that such advice and counselling may have an effect on weight loss, it is inadequate [20]. Limited treatment contact is thought to be the main reason why modest weight loss is achieved [20]. There are available treatment guidelines for primary care, but there are currently few sufficiently effective established treatment offerings available to children and adults struggling with overweight and obesity within primary health care [21]. More extensive treatment in primary care is often randomly organised, at times by local enthusiasts. Treatment is difficult to establish in primary care because of the complexity involved in treating obesity, and while GPs are competent to diagnose obesity, there is a general lack in knowledge about treating the disease [13, 14] and available time [20]. Furthermore, considering coordination between primary and secondary health care providers, integrated care for patients with obesity is underdeveloped [4, 14, 22–24]. In Norway, obese patients are normally referred to secondary health care when they have a Body Mass Index (BMI) ≥ 40 or BMI ≥ 35 kg/m^2^ with complications of obesity, while children should have an iso-BMI > 35 or iso-BMI > 30 kg/m2 with complications of obesity [25]. However, until a patient reaches this stage in the development of the disease, few treatments are available, and when a patient is referred to secondary health care, a lack of coordination and cooperation between primary and secondary care leads to treatments that may be limited in scope and time.

### Education of GP specialists in the health care system in Norway

The health care system in Norway is primarily a public system organised in two levels. Secondary healthcare services are owned and financed by the Ministry of Health and Care Services and managed through four regional health authorities. The primary care level includes general practitioners (GPs), nursing homes, home care services, maternal and child health centres and out-of-hours services. Primary care is organised and financed by the local authorities (municipalities). Even though GPs are organised as a part of the primary care level, GPs are private contractors and not organised in a shared formal organisation that can instruct GPs or act as a partner on behalf of GPs [26].

The educational program for becoming a GP specialist in Norway includes 1 year of practice at a hospital. Currently, there are no positions targeted at GPs’ educational needs, so a GP seeking specialization must apply for a regular specialist training position at a hospital. In most of these positions, the doctor is enrolled in the rota system. This means that candidates will often spend time in emergency admissions on evening and night shifts that are compensated by time off. This leaves little time to attend in-house patients and perform routine follow-up of patients, both of which are relevant for their practice as GPs.

### Theoretical basis

Mur-Veeman et al. [27] have shown that organizational and financial splits between health care providers, such as those present in Norway between primary and secondary health care, hinder integrated care development and delivery. Organizational divides are closely linked to contradictory interests, separate professional cultures, power relations and mistrust between health care providers. Martinussen [28] has shown that the interaction between GPs and hospital physicians has improvement potential, and weak collaboration between GPs and hospitals has been the focus of several studies [29–31]. Delayed or inaccurate communication can have substantial implications for the quality of care, which is especially apparent when patients need lifelong follow-up. Efforts to strengthen integrated care can counteract such inadequate treatment at the interstices between providers. In this paper, integrated care is defined as “[ ] a coherent set of methods and models on the funding, administrative, organizational, service delivery and clinical levels designed to create connectivity, alignment and collaboration within and between the cure and care sectors” [32].

There are many strategies available to foster integrated care. It is found that different commitments, goals and tasks can be major obstacles for collaboration between care levels [33]. Thus, defining roles and having a shared purpose is essential to achieve successful interorganizational collaboration [34]. Other approaches include training of medical staff, a focus on how they perform their responsibilities and tasks, and how they work together with colleagues and patients [32]. Face-to-face interaction is well known to foster trust and collaborative relations. This has also earlier been shown to apply to the relationships between GPs and hospital specialists [28, 35]. Networking and collaboration both horizontally and vertically across health care providers promotes integrated care, as well as a “Shared understanding of patient needs, common professional language and criteria, the use of specific, agreed-upon practices and standards throughout the lifecycle of a particular disease or condition…” [32].

Fruitful integration between care levels is dependent on communication between primary and secondary health care providers [36], and this collaboration becomes even more important for patients with multiple complex conditions and needs [37]. Efforts to improve integration should aim to understand the perspectives of clinicians in each setting and implement strategies that engage both groups by way of shared communication through direct access to each other, interpersonal relations, shared electronic medical records and clearly defined accountability [31]. However, organizational and financial splits between these two parts of the health care system impede such collaboration. The lack of a common hierarchy and governance structure necessitates professionals to create combined responsibilities for shared accountability and decision making to deliver integrated care [38]. There is therefore a need for models and methods that may enhance care delivery suited for patients with complex, long term problems that cut across multiple care providers and settings. Such models should combine the clinical expertise of the specialist and the ability of GPs to bridge the gap between medical and social problems [39] to allow for continuity of care over time. The development of agreed care pathways has the potential to align clinical, management and service user interests across primary and secondary care [40] but has been shown to be most effective in contexts where patient care trajectories are predictable [41]. When pathways are more variable, this is a demanding intervention that requires comprehensive and prolonged efforts by health care professionals in the involved organizations [42].

We have witnessed many efforts to foster integrated care in the past decade, and this topic has received substantial political interest [11, 27]. However, there are few reports on how educational programs for care providers can contribute. A noteworthy exception is Hirsh et al. [43], who studied how a clerkship model may provide undergraduate students with training relevant for the continuity of care. Concerning the specialist training of GPs, there are few examples of similar discussions. Surveying former research in the area revealed that specialist training is rarely debated, and when it is, the discussion concerns evaluation forms, attendance and curricula. We did find a few examples of case studies in which GPs visited local hospitals for knowledge exchange [44, 45]. Such cases have been reported as beneficial for integrated care and mutual learning between GPs and hospital staff, but collaboration lasts a short time, does not involve GPs practising at the hospital and does not demand much involvement between GPs and hospital staff. In response to the challenges described above, the Centre for Obesity Research (ObeCe) at St. Olavs Hospital, Trondheim University Hospital wanted to develop an educational program fostering integrated care. Thus, in 2010, an educational program for the specialization of GPs was established at three regional hospitals in Norway to enhance the exchange of knowledge and strengthen coordination between primary and secondary healthcare providers.

The research question addressed in this paper is thus: what are the main outcomes of the educational program relevant for care delivery to obese patients, as experienced by the participants?

## Methods

### Design

In this study, we investigated how an educational program for GPs in one region in Norway, including one university hospital and two general hospitals, could contribute to enhancing the continuation of care across health care providers. This educational program provides a case study of how educational measures may be designed to promote integrated care. To evaluate the program, understand its potential contribution to integrated care and reveal how it may be improved, a qualitative study [46] was undertaken. Central documents concerning the establishment and organization of the program were read and analysed, and 13 informants were interviewed. All participants in the program, as well as their closest collaborators at the hospitals and a representative from one municipality, were interviewed.

### Research setting

The program was initiated in November 2010 by the management of the Centre for Obesity Research (ObeCe). ObeCe was established in 2005 in line with national and regional health policies [47, 48] and is a research and development centre that has carried out several projects to promote collaboration between primary and secondary health care regarding overweight and diabetes. ObeCe does not have patient treatment as its main concern. This allows the unit the freedom and mandate to create new practices like the educational program for GPs [49]. By June 2014, eight GPs had participated or were currently participating in the project.

The program was designed by ObeCe to provide GPs with relevant training and education for their general practice and to strengthen the connection between primary and secondary care. It was also geared towards providing primary health care providers with competence concerning a grave public health problem, as it focused on subject areas relevant for the prevention and treatment of overweight and obesity. The GPs received extensive training with the same patient groups that they meet in their general practice and which they considered challenging. The costs of the educational program were limited to salary expenses for the involved GPs, in addition, they contributed financially to their respective departments by treating patients.

The GPs were employed in educational positions on temporary contracts limited to one-year full time. They had the possibility to work part time, leaving them the opportunity to continue with their own practice. During the program, the GPs participated both in clinical practice and theoretical studies. They were part of multidisciplinary teams at different departments at the hospital. The program was designed to provide the GPs with knowledge and training in four main areas: clinical practices, theoretical studies, research and the development of integrated care. However, the informants emphasized that the balance between these four areas could be adjusted to allow for individual interests and needs. A tutor supported them during the course of the program.

The GPs worked at three different departments to gain clinical practice: the Multidisciplinary Outpatient Clinic for Obesity, the Department of Endocrinology and ObeCE. The multidisciplinary Outpatient Clinic for Obesity is a clinic with health professionals such as surgeons, psychologists, nutritional experts, physiotherapists and endocrinologists that receives children, adults and families, and has a broad perspective on obesity-related issues similar to the perspective of a GP. The GPs did not participate in the rota system, so they had fixed days each week at the different departments: three days at the Department of Endocrinology, one day at the Multidisciplinary Outpatient Clinic for Obesity and one day at ObeCe. They were given their own list of patients to follow for an extended period.

They were involved in patient care at the clinics and in preparing obese patients for operations and for self-management. They also received training in treating patients with type 2 diabetes at the Department of Endocrinology. The GPs evaluated referrals to the hospital from other GPs, conducted physical examinations, reviewed medical histories, provided diagnoses and followed up with tests. These examinations provided the basis to determine the severity of the disease and to create a treatment plan for the patient. They followed patients long enough to observe the effects of the treatments and the patients’ experiences.

The educational program was devised to give the GPs access to theoretical studies and they participated in courses, both initiated by the hospital in general and available at each clinic. Internal courses at the hospital (two hours each week) are required for any specialist under training, but in this program, the GPs also attended lectures at each clinic for one hour each week. In addition, they contributed themselves by holding lectures for the staff at the clinics and at ObeCe. They were also given leeway and encouraged to take initiative in areas they felt they could improve and contribute to.

The GPs had 20% of their time dedicated to research and were encouraged to contribute to the professional development of the field of obesity and overweight. They were expected to update themselves on the latest research results, and as a requirement of the program, the GPs participated in on-going research projects within one month of their employment. The GPs who participated in the program received their formal qualifications as specialists and re-certification in line with the purpose of the program.

### Participants

All those involved or related to the program at the time of the study and available for interviews were asked to participate, and all accepted. Those interviewed included five GPs who were enrolled in the program, two nurses, one research assistant, four head physicians and one manager from the local municipality, thereby covering those informants most involved and familiar with the project. Interviews were conducted in two rounds: in April 2012 and in September 2013. The methodological approach was deemed appropriate, as the study was exploratory and aimed to uncover respondents’ experiences and opinions. Each interview lasted from one to two hours. Participation was voluntary.

### Data collection and analysis

As a basis for the interviews, a semi-structured guide was developed reflecting the research questions of the study and sent to each informant before the interview (see Additional file 1, Interview guide). Informants were asked how they experienced the program, in particular about the effects of the program on their own knowledge, expertise and ability to provide quality of care, as well as the results for the various clinics at the hospital and the primary health care services. Informants were also asked whether they saw the program as useful for the particular patient group and society at large, and if so, in what ways. The guide was flexible and allowed the informants to include any new themes they found relevant to describe the program and their experience. All interviews were taped and later transcribed. The data was systematically categorised and coded. The analytical process focused on identifying and differentiating the concepts and topics the informants described, both those introduced by the interview guide and those provided by the informants themselves. Concrete examples of integrated care practices and other examples of results from the program were noted. Similarities and differences between each informant and the informant’s group were coded and compared. The analytical approach was inductive and exploratory, focusing on the concepts and categories as described by the informants.

## Results

In interviews, both GPs and their collaborators at the hospital emphasized three main areas they saw as important outcomes of the program: (1) the establishment of relations and networks which breached the organizational divide between primary and secondary care; (2) increased knowledge and competence both at the primary care level and at the hospital; and (3) the development of shared practices and the use of shared standards. In addition, the informants also identified shortcomings, mainly related to the weak integration the GPs experienced with their own municipalities.

### Establishing relations and networks

Through the program, GPs and hospital staff became acquainted and formed collaborative relations, both during the time the GPs were at the hospital and after. Since the GPs worked in three different departments at the hospital, they were able to establish relations with several colleagues at the hospital, both doctors and nurses. Both GPs and hospital staff emphasised in interviews the value of the personal relations and networks they had been able to build through the program. One of the GPs stated:

“The most important thing is the increased competence and the network of contacts you get. It becomes so much simpler. And that is a part of the point, that it becomes seamless and that it should work like this” (GP3).

The GPs were encouraged to contribute to areas they felt they could improve and, as a result, they established the first formal arena for knowledge exchange between staff at the ObeCe and the Outpatient Clinic for Obesity so that employees from the two departments can meet at regular intervals for lectures and research updates. This was made possible because the GPs worked fixed days at both departments and were, unlike their colleagues at the hospital, not in the rota system. The rota system affords less individual predictability, as it is not known well in advance which person will occupy which shift. The GPs would know months in advance where they would work each day, so it was easier to plan ahead and take responsibility to schedule activities for knowledge exchange with their hospital colleagues.

The program aided in forming new relations and networks. The GPs emphasised in interviews how this was made possible because they were met by informed and positive colleagues at the hospital. Respect and trust characterised the relations that were established and laid the grounds for open discussions and mutual learning.

### Increased knowledge and competence

The program offered several opportunities for training, both through formal courses initiated at the hospital and through the time specified for research. The interviewed GPs explained that they had increased their knowledge of overweight and obese patients, and related diseases such as diabetes, through participating in the program.

According to the informants, the professional environment of the hospital, the time set aside for research and the tutoring they received during the program gave room for investigations that the GPs rarely had time for in their general practice.

The program also provided knowledge and competence that extends beyond those of a formal qualification in overweight and obesity treatment. In an interview, one of the participating GPs explained how the program had changed her attitude towards the patient group and increased her understanding of the complexity of the field of overweight and obesity:

“It is easy to have prejudices concerning this patient group, and there is much stigmatisation. I notice that with colleagues and others who are not familiar with the field. It is easy to conclude, as with other lifestyle related diseases, that it is self-inflicted and weak individuals who are not capable of changing their own situation. It is of course not so simple” (GP1).

The GPs also stated they had an increased sense of confidence in treating obese and overweight patients with related diseases such as diabetes when returning to their medical practices. During their time at the hospital, they were able to see a large number of patients with type 2 diabetes, which increased their confidence in their ability to treat this patient group. Earlier, they would have referred type 2 diabetes patients to secondary health care because they did not feel confident enough to treat them themselves. As explained by GP2:

“My attitude changes while I am here [at the hospital]. I see possibilities for all that can be done in my general practice. Before, I would think that ‘now I have tried with this patient for half a year, and nothing happens, we won’t get any further.’ Now, with new knowledge, I believe there is more we can do” (GP2).

There was a mutual exchange of knowledge between the GPs and medical personnel at the hospital, especially concerning care delivery. A head physician at one of the clinics where the GPs worked said:

“Professionally, it has been very positive for our clinic that they have brought with them the GPs’ views into treatment for diabetes. We are able to discuss what is feasible to do in a general practice, and what we need to continue to do here” (Head Physician).

### Shared practices and use of shared standards

It was stated in the objectives of the program that the GPs were to contribute to increased cooperation between the primary and secondary health care levels. The program had thus allocated funds for arranging conferences and meetings between the GPs and their respective municipalities.

The program resulted in the dissemination of knowledge not only to the participating GPs, but also to health personnel at the primary care level. Several of the GPs initiated training courses and lectures for fellow GPs in their municipalities that occurred at their own medical centres, in larger conferences and in permanent colloquium groups where a smaller number of GPs met regularly for research updates and discussions. One of the projects in which the GPs were involved investigated how an intermediate care service at the primary health care level could assume the postoperative follow-up of obese patients in collaboration with local GPs.

An internet course qualifying GPs for their specialisation was developed by one of the GPs in the program and launched nationwide. Also, general information leaflets were developed, providing information to GPs concerning the treatment of patients who have undergone gastric bypass operations. This included a document listing the short- and long-term side effects of gastric bypass. The involvement of the GPs at ObeCe also resulted in several international publications written by them and their colleagues at the hospital, thus disseminating knowledge to a broader international audience.

In the clinical domain, the program resulted in improved shared understanding and practices between the primary and secondary health care levels. This was achieved by the close involvement of the GPs at different departments in the hospital, and through the activities the GPs initiated at their own medical centres and in primary care in general. The GPs said they had realised that through their knowledge and experience of both primary and secondary health care, they could play an important role in creating shared practices across care providers.

Shared practices between primary and secondary care were developed and continuously improved because of the program. These measures allowed for the increased involvement of and information to GPs when their patients were at the hospital. One of the nurses summed up the new practices for the GPs’ strengthened involvement:

“[GP1] has done much for communications with the GPs. Patients receive a letter, and the GPs get notified that the patient is in a post-operative group. Patients receive a form they need to fill out. This didn’t exist before [GP1] and the other GPs were here” (Nurse 1).

Also, specific procedures for follow-up of patients who had undergone gastric bypass were amended, as they did not function properly. Patients had earlier failed to attend their appointments at the hospital and the patients’ GPs were not involved. Also, in the case of diabetes patients, the primary care level was not actively involved. Alternate consultations between the hospitals and respective GPs were therefore initiated as a result of the program. This was seen as a way to enable the primary health care level to be more involved in follow-up care. In addition, the program resulted in initiatives to improve horizontal integration across health services, social services and other care providers.

Both physicians and nurses said they gained much knowledge through the close contact with GPs. They emphasised having an increased understanding of the GPs’ opportunities and constraints when attending patients in their general practices. Hospital staff also claimed that they now recognised the need to collaborate more closely with GPs while their respective patients were receiving treatment at the hospital and after, and that they understood more of how to improve the collaboration between primary and secondary health services. Some also said they had changed their practices as a result of what they learned from the GPs.

The informants perceived that the educational program had immediate value for those involved in enabling and supporting the development of integrated care. It provided high quality training for GPs while meeting the national and local challenges of achieving integrated care. One of the GPs concluded:

“The project has been successful, because we have become a part of the work at ObeCe, meaning integrated care in practice, because we are GPs playing on the same field as the secondary health care services” (GP5).

### Shortcomings

However, the program also had shortcomings. These concerned the relationship and collaboration between the GPs and their respective municipalities. One of the reasons given by the respondents was that the organisation of the municipalities’ services and the decision-making process is fragmented. The interviewed GPs were unsure of who they could present ideas to at the municipality to initiate projects they believed could improve health care services and did not know whether their ideas would be in line with current plans and budgets. One stated:

“Yes, I am sitting here and I want to make things happen in my municipality, but it is not so easy when there are no systems or frameworks for it. You have to make it happen yourself, and nobody expects anything” (GP2).

The GPs found it difficult to know whom to approach at the municipalities and how they could work to realise their ideas. The informants suggested that the educational program should include clear expectations for how the GPs could establish plans for their work in the municipalities while under training, because when they returned to their general practices, there would be less time to plan and think about new projects.

## Discussion

### Outcomes of the program

The educational program incorporated several strategies earlier identified as beneficial for fostering integrated care in three important domains: organisational, service delivery and clinical practice [32]. Central to the development of integrated care is vertical integration between primary and secondary health care through **formal and informal relations, networks and collaboration**, which breaches the organizational divide between the two systems [27]. High quality service delivery hinges on the **knowledge and competence** of medical staff both at the primary and secondary health care levels, and is not only related to the specific disease(s), but also to care delivery [32]. In the clinical domain, a shared understanding of patient needs and use of **shared practices and standards** between providers is essential [37]. Interviews with personnel involved in the program indicate that the program showed results in these directions, even though there were also shortcomings. For example, the interviewed GPs did not know whom to approach in their respective municipalities to realize new ideas and changes in care delivery.

The educational program has been shown to be able to foster relations between hospital staff and GPs, which are lacking in the existent health care system. This is important for a patient group that will continuously be in need for care to avoid serious complications and that has the risk of becoming revolving-door patients due to a fragmented and poorly integrated health care system. As Tricco et al. found [50], multidisciplinary care is needed for chronic patients with complex conditions, and improving care for this group is effective at reducing readmissions. Care needs to be provided in a continuous interplay between primary and secondary care by health professionals who have defined roles and responsibilities and a shared purpose [31, 33, 34]. This corresponds to earlier recommendations for care delivery for patients with complex care needs [37], that shows that their need for care is best met by close interaction and collaboration between primary and secondary health care providers.

The educational program contributes to integrated care for obese patients by combining the expertise of specialists from the hospital with the broader and more holistic experience and competence of GPs [39]. Obesity is a condition that requires caregivers to bridge medical and social problems. With the increased prevalence of complex conditions, hospitals cannot simply discharge patients to primary health care without themselves offering to share their knowledge and expertise. Secondary health care has experienced a strong increase in referrals for patients suffering from obesity and related conditions. To reduce this burden, secondary health care providers need to engage with care givers in primary care to strengthen their ability and capacity to treat this patient group. Also, medical staff at secondary health care institutions need to gain an understanding of how obesity and subsequent treatment are intertwined with broader issues such as work, family life and social problems, as well as the framework conditions of the patients’ local communities. A Cochrane review [51] concluded that audit and feedback strategies can be important to improve professional practice, but this improvement depend on how the feedback is provided and by whom. Creating a learning environment, as in this educational program where health professionals openly discuss practices and alternative approaches, can thus be a potential strategy for enhancing quality of care.

Through collaboration and direct dialogue, the GPs and specialists involved in the educational program create and shape shared understanding and practices. Patient’s awareness of such dialogues between the GP and specialists from the hospital has earlier been suggested to strengthen patients’ sense of security [52]. Collaboration between caregivers from primary and secondary health care services is important both for the quality of care that is given to this patient group and to ensure continuity of care. Other strategies, such as developing agreed care pathways, could provide stronger alignment between primary and secondary health care providers, but it might also demand much efforts and prolonged engagement to implement [42], especially when patient pathways are variable [41], as in the case of obesity.

The educational model thus promises to compensate for some of the problems of the current organization of the health care system [27]. The artificial division between clinical specialists at hospitals and GPs in primary care has earlier been shown to lead to weak communication, which affects the continuity and quality of care [28–31]. A doctoral thesis concluded that integration depends on the collaborative partners’ ability to develop all-embracing objectives and view their services and work as a part of the total chain of care. Integration depends on sufficient communication and interorganisational teamwork, a learning environment, common perspectives and clarified roles [36]. Through developing relations, enhancing knowledge and competence and shared understanding and practices, the educational program studied here promises to breach obstacles to continuity of care for patients suffering from obesity. There are many different strategies that have been shown to be conducive to enhancing quality of care, both within and across primary and secondary care. However, as Grol and Grimshaw [53] argue, approaches should be fit for purpose and adapted to the barriers and facilitators to change in each situation. The approach chosen here answered a perceived need to strengthen knowledge in primary care. The program continues and is now in use at several departments at St. Olavs Hospital. It should be considered a step towards strengthening integrated care between primary and secondary care.

In Central Norway, since 2010, the educational program has gradually been instituted as a permanent program that is offered to a number of departments at St. Olavs Hospital. As of today, 35 GPs have been employed at seven different departments (Department of Ophthalmology, Department of Ear, Nose and Throat, Head and Neck Surgery, Child Department, Department of Endocrinology, Department of Neurology, Department of Clinical Pharmacology and Department of Gynaecology) at St. Olavs Hospital, Trondheim University Hospital, and in three different departments (Department of Geriatrics, Child Department and Department of Surgery) at Namsos Hospital. According to the Director of Integrated Healthcare at St. Olavs, the experiences from these departments are univocally positive (Personal communication, email to author BK, 6.1.2018). In sum, these experiences reflect this study’s findings. The departments found it useful to interact with GPs to learn more about the expertise in general practice and the GPs increased their knowledge, which in turn was transferred to their colleagues in primary care. Increased knowledge and competence in primary care resulted in fewer referrals to the hospital. Finally, the capacities of the different departments at the hospital increased with the aid of the GPs.

The educational program did not seek to alter the organizational divides between primary and secondary care, but focused on strengthening connectivity and collaboration across these divides by involving GPs in secondary care for a defined time period. According to the informants, this had positive impacts for both primary and secondary care, as discussed above. This was considered a necessary first step to demonstrate the usefulness and feasibility of the program, considering the large number (47) of municipalities in this region, all with highly diversified tasks and structures. An important lesson learnt from this program is that while obesity and diabetes are a growing concern in Norwegian municipalities, it is important to designate funding of assigned positions directed towards such illnesses as a cost-sharing scheme across several municipalities. The continuation of the program now (2018) shows that groups of municipalities are engaging with secondary health care providers to incorporate the increased knowledge and experience of the GPs in municipal structures.

### Limitations and future research

The empirical basis for this article is limited, with 13 respondents and one case, although it was carried out in three hospitals. The results are indicative of how such an educational program may contribute to integrated care, but a more extensive program and more studies are needed to reach findings that can be considered representative. The strength of the study is its reporting of a novel model that may foster integrated care and strengthen the expertise of primary care while reducing the burden on the acute sector.

Revising educational programs in line with the model described here may be an affordable and feasible approach to dealing with some of the organizational splits between health care providers. The costs of the program were limited to salary expenses for the participating GPs, and their work at the hospital contributed financially to the respective departments. However, further research should also assess the costs of the intervention and compare these to other strategies for integration. Nevertheless, there is a need for more systematic knowledge of how educational programs may contribute to integrated care and how such programs may have long-term effects on the collaboration between primary and secondary health care providers. Further research should study the effects of such programs, and especially seek to assess how patients experience strengthened interactions and collaboration between GPs and hospital staff.

## Conclusion

The educational program illustrates how one may combine high quality training with integrated care. It constitutes a promising path for both increased medical competence and improved integrated patient care because it involves health care personnel from both primary and secondary care who together develop practices that are implemented across care providers. The program is applicable to different professional domains, especially those where patients can benefit from coordinated health services and where health personnel can collaborate to develop practices that merge competences and approaches from both primary and secondary health care services.

Important challenges remain in engaging more municipalities to incorporate the increased knowledge of GPs into municipal structures and to disseminate the lessons learnt from this program to other regions.

## Additional file

Additional file 1: Interview guide. (DOCX 24 kb)

## Abbreviations

BMI: Body Mass Index
GP: General practitioner
ObeCe: Centre for Obesity Research
