# Supplementary material for: Enhancing knowledge and coordination in obesity treatment: a case study of an innovative educational program
Source: BMC Health Serv Res. 2019 May 2;19:278. doi: 10.1186/s12913-019-4119-9 (PMC6498688; doi:10.1186/s12913-019-4119-9)
Supplement: Supplementary file 1 — Interview guide. (DOCX 24 kb) [file 12913_2019_4119_MOESM1_ESM.docx]

**Interview guide**

**Evaluation of the educational program «GP in interdisciplinary polyclinic»**

The interview is part of an evaluation of the program, and focuses on three main areas; practical implementation, what benefits have been achieved, and opportunities for improvement in the future. Results from 12-14 interviews will be summarized in a short evaluation report and be used for a research article. In line with good research ethics, participation in the interview is voluntary, and results will not refer to individual participants. It is important to be aware that with such a small selection of informants it will be possible to recognize quotes and statements referred to, for someone who knows the respondents and the group well. It is therefore possible to reserve yourself against the use of direct quotes from the interview.

The interview will take 1 - 1.5 hours, and after consent will be recorded on tape. Recordings will be stored on a secure server and deleted immediately after the evaluation project is completed.

For further information, please contact Tonje C. Osmundsen, at tonje.osmundsen@samfunn.ntnu.no, tel. 98217758.

**Expectations**

Which expectations did you have to the program at the onset?

What did you perceive as strengths and benefits of the program?

**Implementation**

In what manner did you become/are you involved in the program?

How did you experience the implementation of the program?

- Access to information beforehand
- Organizational structure (employment, involvement of new employees, access to data systems etc.)
- Judicial framework
- Financial instruments
- Professional framework (training, collaboration with colleagues etc.)

**Benefits and effects**

What have you experienced as effects of the program?

- Increased knowledge and understanding
- Increased ability to perform your tasks
- Benefits for you own career (specialization, other?)
- How would you/or is it possible to estimate the effect of these benefits?

What benefits do you perceive for your department?

- Increased knowledge
- Capacity
- Work environment
- How would you/or is it possible to estimate the effect of these benefits?

In what manners is or will the project be useful for patients, or other groups in society?

**Improvement potential**

What could have been improved?

Are there possible use values in this program which are not fully explored today?

What needs to be done differently to improve the program?

Has the program and its implementation been in line with the expectations you had at the onset?

If you reflect on the program in a greater societal context (for instance related to the Coordination Reform) – in what manners could/should the project be expanded, be combined with other instruments, increase use value for others, or be changed in other ways?
